# Hydroxyl radical-aided thermal pretreatment of algal biomass for enhanced biodegradability

**DOI:** 10.1186/s13068-015-0372-2

**Published:** 2015-11-26

**Authors:** Le Gao, Demao Li, Feng Gao, Zhiyong Liu, Yuyong Hou, Shulin Chen, Dongyuan Zhang

**Affiliations:** Tianjin Key Laboratory for Industrial BioSystems and Bioprocessing Engineering, Tianjin Institute of Industrial Biotechnology, Chinese Academy of Sciences, No. 32, Xiqi Road, Tianjin Airport Economic Park, Tianjin, 300308 China

**Keywords:** Pretreatment, Hydroxyl radical-aided thermal pretreatment, Algal biomass, Hydroxyl radicals, Fenton system

## Abstract

**Background:**

Algal biomass, known as a potential feedstock for biofuel production, has cell wall structures that differ from terrestrial biomass. The existing methods for processing algae are limited to conventional pretreatments for terrestrial biomass.

**Results:**

In this study, we investigated a novel hydroxyl radical-aided approach for pretreating different types of algal biomass. In this process, hydroxyl radicals formed by a Fenton system were employed in combination with heating to alter the crystalline structure and hydrogen bonds of cellulose in the algal biomass. FeSO_4_ and H_2_O_2_ at low concentrations were employed to initiate the formation of hydroxyl radicals. This method releases trapped polysaccharides in algal cell walls and converts them into fermentable sugars. The effects of temperature, time, and hydroxyl radical concentration were analyzed. The optimal pretreatment condition [100 °C, 30 min, and 5.3 mM H_2_O_2_ (determined FeSO_4_ concentration of 11.9 mM)] was identified using a central composite design. Complete (100 %) carbohydrate recovery was achieved with some algal biomass without formation of inhibitors such as hydroxymethylfurfural and furfural as by-products. Both microalgal and macroalgal biomasses showed higher enzymatic digestibility of cellulose conversion (>80 %) after the milder pretreatment condition.

**Conclusion:**

Hydroxyl radical-aided thermal pretreatment was used as a novel method to convert the carbohydrates in the algal cell wall into simple sugars. Overall, this method increased the amount of glucose released from the algal biomass. Overall, enhanced algal biomass digestibility was demonstrated with the proposed pretreatment process. The new pretreatment requires low concentration of chemical solvents and milder temperature conditions, which can prevent the toxic and corrosive effects that typically result from conventional pretreatments. Our data showed that the advantages of the new pretreatment include higher carbohydrate recovery, no inhibitor production, and lower energy consumption. The new pretreatment development mimicking natural system could be useful for biochemical conversion of algal biomass to fuels and chemicals.

**Electronic supplementary material:**

The online version of this article (doi:10.1186/s13068-015-0372-2) contains supplementary material, which is available to authorized users.

## Background

Algal biomass, known as a “third-generation biomass feedstock” for biofuel production, has received considerable attention because of its high productivity, negligible lignin content, and high CO_2_ mitigation effects compared with terrestrial biomass [1]. Algal biomass processing for biofuel production also has other advantages such as low lignin and hemicellulose concentrations compared with other lignocellulosic plants [2]. Compared with land plants, algae have different cell wall layers with different chemical compositions but lack an overall generic structure. Algal cell walls are thin, and their primary and secondary cell walls are undivided [3]. An algal cell wall without lignin has a relatively loose structure, which is different from other plant cell walls. The hydrolysis of algae to produce fermentable sugars aside from high-value products, however, often hinders the comprehensive utilization of algae. The algae pretreatment is regarded as an important step to facilitate the release of the sugars [4]. The pretreatments for algal biomass are typically performed under extreme conditions: high temperatures of 120–220 °C, and high concentration of chemical solvents to increase relative content cellulose, which still followed the traditional pretreatment methods for terrestrial biomass. There have been many reports about algal pretreatments. The marine macroalga *Nizimuddinia zanardini* was subjected to 7 % dilute sulfuric acid pretreatments at 121 °C for 45 min, and the cellulose conversion of macroalgae was only 31.8 % [5]. An acid pretreatment using 0.06 % (w/w) sulfuric acid at 170 °C for 15 min was employed to *Saccharina japonica*, brown macroalgae. The cellulose conversion of macroaglae reached 83.37 % [6]. Numerous inhibitors, such as hydroxymethylfurfural (HMF), 2-furfural, and acetic acid, were also formed in hydrothermal pretreatment at 200 °C, wet oxidation pretreatment using 12 bars of O_2_ at 200 °C, and steam explosion pretreatment [7], besides acid or alkali pretreatment for algal biomass. These inhibitors may affect the downstream production of high-value algae products. A neutralization process is required for enzymatic hydrolysis after dilute acid pretreatment. Current algae pretreatments are very expensive because of their high-temperature and high-energy requirements. A new pretreatment technology is urgently needed for algal comprehensive utilization with low cost and low energy.

In nature, the wood-decaying microorganisms and wood-feeding termites are able to digest lignocellulosic substrates efficiently using hydroxyl radicals [8]. The lignocellulosic-preconditioning system used hydroxyl radicals to depolymerize lignocellulose under mild environmental conditions. A well-known process that produces radicals is Fenton’s reaction in which hydroxyl radicals (·OH) are produced in the solution through the reaction of Fe^2+^ ions with H_2_O_2._ Using Fenton reaction, hydroxyl radicals formation was simulated and amplified in vitro. There has been report on use of ·OH to pretreat lignocellulosic biomass [9]. According to Gould [10], lignocellulosic biomass requires a long pretreatment time, relatively harsh conditions, and a high ·OH concentration because of the relatively tight structure of lignocellulose.

$${\text{Fe}}^{2 + } + {\text{H}}_{2} {\text{O}}_{2} \to {\text{Fe}}^{3 + } + {\text{OH}}^{ - } + \cdot {\text{OH}}\, ( {\text{Fenton's}}\,{\text{reaction)}}$$

For the unique structural features of algal biomass, this study investigated a new pretreatment based on hydroxyl radicals’ effects of processing under the milder reaction conditions and low concentrations of chemical reagents. The present study investigated the effects of processing variables, including hydroxyl radical concentration, pretreatment time, and pretreatment temperature, on the glucose production of different types of algal biomass. Moreover, the present study explored good applicability of the new pretreatment for different algal biomasses containing four macroalgae (*Macrocystis pyrifera*, *Ulva prolifera*, *Gelidium amansii*, and *Porphyra umbilicalis*) and four microalgae (*Chlorella sorokiniana*, *Scenedesmus quadricanda*, *Haematococcus* *pluvialis*, and *Chlamydomonas hedleyi*). The hydroxyl radical-aided thermal pretreatment achieved complete (100 %) carbohydrate recovery of algal biomass and substantially improved algal biomass digestibility. There were no inhibitors produced in this pretreatment process. This new pretreatment is a low-cost, high-recovery, and environmentally friendly pretreatment method, which could combine with other high-value materials’ extraction from algal biomass to achieve the complete utilization of alga.

## Results and discussion

### Algal biomass content

The constituents of the algal biomass significantly differed from those of terrestrial biomass and also varied with species. The principal components of algal cell wall are listed in Table 1. Green algae contained the highest lignocellulose content. The total amounts of cellulose and hemicellulose in *U. prolifera*, *C. sorokiniana*, *S. quadricanda*, *H. pluvialis*, and *C. hedleyi* were 18.81, 22.69, 16.86, 30.97, and 20.12 %, respectively. The cellulose content in green algal biomass was higher than that in red and brown algal biomasses. Cellulose is a partially crystalline skeletal component that provides strength. The liquid contents of the microalgal biomass from *C. sorokiniana*, *S. quadricanda*, and *C. hedleyi* were similar. The biomass from red algae, unlike those from the other algae, had higher hemicellulose content and lower ash content. The hemicellulose contents in red algal biomass, including 47.16 % in *P. umbilicalis* and 24.37 % in *G. amansii*, were higher than those in the other biomass. The ash contents in *C. sorokiniana* and *H.* *pluvialis* were approximately 3.79 and 3.93 %, respectively, each of which was one tenth of the ash content in brown macroalgae. High ash content is not conducive to the effect of pretreatment because it causes slagging and fouling problems during thermochemical conversion [11]. The principal polysaccharides in brown algae are alginates, laminarin, and mannitol [12], whereas those in red algae are agar and carrageenan [13]. The biodegradability of each component has not been fully investigated. Besides the high-value products from algal biomass, the algal cell contains significant quantities of carbohydrates, which may be used to produce glucose for biofuels. In this connection, choosing the correct method for algal biomass pretreatment is obviously important, since it affects the enzymatic digestibility of algal biomass. At the next stage of this work, the pretreatment condition of algal biomass would be evaluated.

**Table 1:** Experimental design matrix for the optimization of pretreatment conditions

Run	Tri no.	Tri no.	Temperature (°C)	Time (min)	H_2_O_2_ concentration (mM)
1	J1^a^	H1^b^	80	30	7.1
2	J2	H2	120	15	5.3
3	J3	H3	120	30	7.1
4	J4	H4	100	15	7.1
5	J5	H5	100	45	7.1
6	J6	H6	80	30	3.5
7	J7	H7	100	30	5.3
8	J8	H8	120	30	3.5
9	J9	H9	100	30	5.3
10	J10	H10	100	15	3.5
11	J11	H11	100	45	3.5
12	J12	H12	80	15	5.3
13	J13	H13	100	30	5.3
14	J14	H14	80	45	5.3
15	J15	H15	120	45	5.3

^a^ *Macrocystis pyrifera*

^b^ *Ulva prolifera*

### Effect of pretreatment conditions on enzymatic hydrolysis

**Fig. 1:**
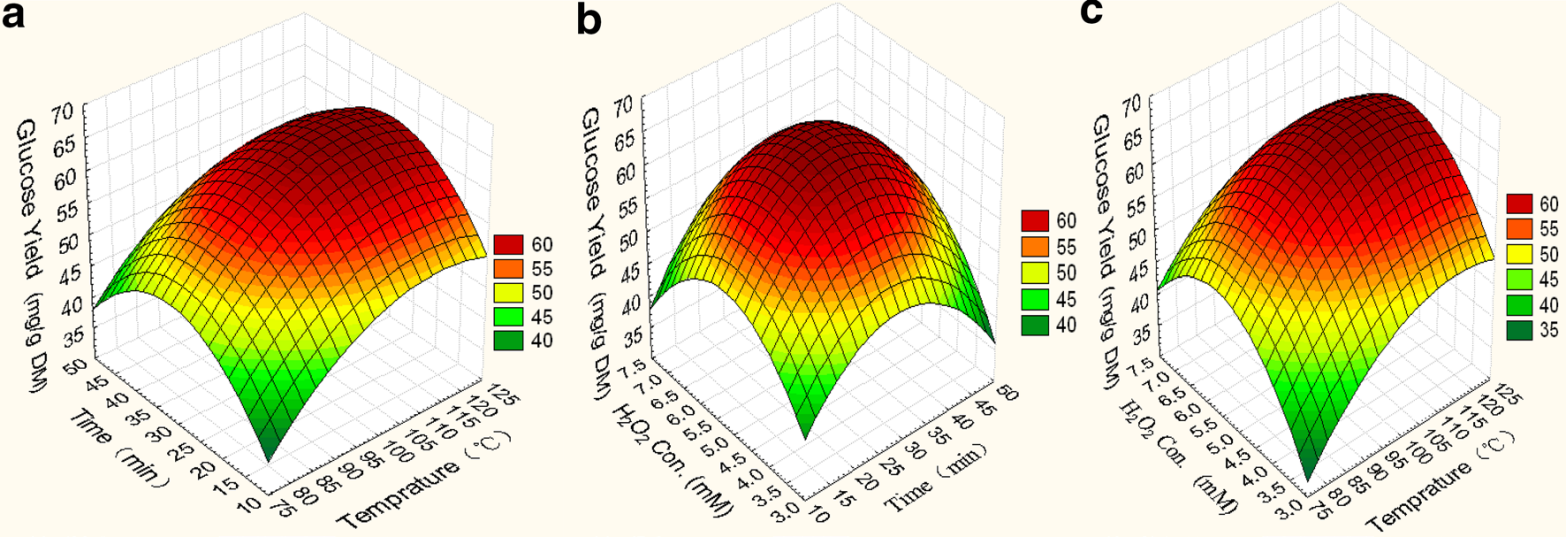
Response surface curve representing the interactive effects of temperature, time, and FeSO_4_ concentration on the digestibility of *Macrocystis pyrifera*: **a** effects of temperature and time; **b** effects of temperature and H_2_O_2_ concentration; and **c** effects of time and H_2_O_2_ concentration

**Fig. 2:**
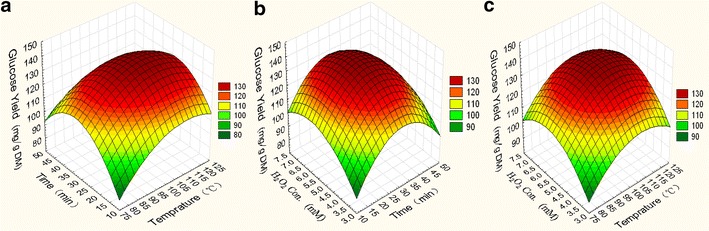
Response surface curve representing the interactive effects of temperature, time, and FeSO_4_ concentration on the digestibility of *U. prolifera*: **a** effects of temperature and time; **b** effects of temperature and H_2_O_2_ concentration; and **c** effects of time and H_2_O_2_ concentration

Table 2 presents the effects of hydroxyl radical-aided thermal pretreatment on the glucose yield of different types of algal biomass, including *M. pyrifera* and *U. prolifera*, under different reaction times, temperatures, and H_2_O_2_ concentrations. The optimal pretreatment conditions for *M. pyrifera* and *U. prolifera* were 100 °C, 30 min, and 5.3 mM H_2_O_2_ (Trial Nos. 7, 9, and 13 in Table 2). The glucose yields from *M. pyrifera* and *U. prolifera* were 64.63 and 143.19 mg/g dry matter (DM) (average of J7, J9, J13), respectively (Figs. 1, 2). The cellulose conversions in *M. pyrifera* and *U. prolifera* reached 98.59 and 84.79 %, respectively, which were higher than those obtained using conventional pretreatment methods [14]. Meanwhile, under 1 % alkali or 1 % acid pretreatment for 60 min, the *M. pyrifera* cellulose digestibility only reached 62.2 and 54.1 %, respectively. The glucose yields from macroalgae that were pretreated with 0.5, 3.5, and 7 % dilute sulfuric acid at 120 °C and then subjected to commercial cellulose conversion are 16, 19, 31.8 %, respectively [14]. The cellulose conversion of the brown macroalga *Sargassum* sp. reached 21.6 % after treatment with 5 % H_2_SO_4_ at 115 °C for 90 min and then hydrolysis using a high load of commercial cellulase (50 FPU/g of biomass) [14]. The *M. pyrifera* cellulose digestibility reached 92.1 % under integrated hydroxyl radicals and hot water (HW) pretreatment which needs two-step process with an HW pretreatment as step I and an HR pretreatment by Fenton reaction as step II [15]. The hydroxyl radical-aided thermal pretreatment in this study integrated two steps of previous pretreatment to form a simple technology and used a high temperature of hot water pretreatment to accelerate the motion of hydroxyl radicals, which had better effect on enhancing algal biomass than the previous pretreatment.

**Table 2:** Proximate analysis of dried algal biomass

	Microalgae				Macroalgae			
	*Chlorella sorokiniana*	*Scenedesmus quadricanda*	*Haematococcus* *pluvialis*	*Chlamydomonas hedleyi*	*Ulva prolifera*	*Porphyra umbilicalis*	*Gelidium amansii*	*Macrocystis pyrifera*
Cellulose (%)	17.69 ± 0.078	16.04 ± 0.032	16.55 ± 0.048	18.67 ± 0.041	15.20 ± 0.024	9.84 ± 0.011	9.09 ± 0.013	5.90 ± 0.010
Hemicellulose (%)	4.99 ± 0.014	0.82 ± 0.049	14.42 ± 0.034	1.45 ± 0.012	3.61 ± 0.014	47.16 ± 0.057	24.37 ± 0.044	0.91 ± 0.010
Ash (%)	3.79 ± 0.017	21.25 ± 0.042	3.93 ± 0.012	30.27 ± 0.047	26.10 ± 0.033	6.77 ± 0.012	4.89 ± 0.012	35.2 ± 0.057
Crude fat (%)	23.74 ± 0.024	23.10 ± 0.041	11.25 ± 0.048	24.75 ± 0.041	3.70 ± 0.011	9.75 ± 0.022	7.75 ± 0.013	4.33 ± 0.011
Protein (%)	2.74 ± 0.011	2.23 ± 0.012	3.56 ± 0.016	1.66 ± 0.012	2.70 ± 0.017	1.83 ± 0.018	2.80 ± 0.015	0.29 ± 0.001
Others (%)	47.05 ± 0.047	36.56 ± 0.043	50.29 ± 0.054	23.20 ± 0.022	48.69 ± 0.041	24.65 ± 0.057	51.10 ± 0.057	53.37 ± 0.049

Less or more stringent pretreatment conditions resulted in low conversion ratios of cellulose. As shown in Table 1, temperatures up to 120 °C produced low glucose yields, which were also observed in other pretreatment methods. Grohman et al. [16]. reported that acid pretreatment temperatures between 140 and 160 °C produce highly digestible cellulose from aspen and straw biomasses. High acid pretreatment temperatures up to 180 °C significantly decrease the amount of released fermentable sugars. Many studies suggested that 140 °C is the most suitable acid pretreatment temperature [17]. Hydroxyl radical-aided thermal pretreatment, which requires a temperature of 100 °C for 30 min, is milder than conventional pretreatments. Therefore, the pretreatment used in this study is more energy efficient.

The pretreatment method used in the present study requires lower concentrations of chemical solvents than acid or alkali pretreatment. The costs of concentrated acid for the dilute acid pretreatment, O_2_ for the wet oxidation pretreatment, energy consumption in the steam pretreatment, and solvents in the organic solvent pretreatment were too expensive compared with the value of glucose [15]. The lower processing temperature, fewer chemicals, and enzyme dosage of the new pretreatment indicate that it is likely to cost less than conventional methods. The material cost of the new pretreatment was 13.17 US dollars/ton biomass, which contained the cost of ferrous sulfate, hydrogen peroxide, and water. The low-pressure steam cost of new pretreatment was 9.35 US dollars/ton biomass. The cost of this new pretreatment for one ton of algal biomasses was 22.52 US dollars/ton biomass, which is 1/3 of the traditional pretreatment cost. The new pretreatment does not need corrosive liquid and a neutralization process, which could reduce equipment requirement and equipment depreciation.

### Applicability of hydroxyl radical-aided thermal pretreatment for different types of algal biomass

**Fig. 3:**
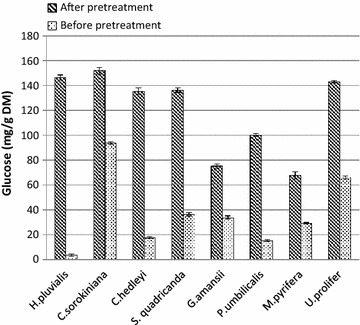
Comparison of the enzymatic digestibility of algal biomass before and after thermal hydroxyl radical pretreatment

As shown in Fig. 3, eight types of algal biomasses, including four from microalgae and four from macroalgae, were processed under the optimal experimental conditions of 100 °C, 30 min, and 5.3 mM H_2_O_2_. The untreated and treated algal biomasses were compared by enzymolysis experiment to analyze algal digestibility change. The glucose yields of the microalgae *C. sorokiniana*, *S. quadricanda*, *H. pluvialis*, and *C. hedleyi* after saccharification were 152.07, 136.45, 146.43, and 136.65 mg/g DM, respectively, which were equivalent to approximately 80 % algal cellulose conversion. The values were approximately 0.62-, 2.72-, 39.64-, and 6.67-fold higher, respectively, than that from untreated algal biomass. The effect of this pretreatment was better on macroalgae than on microalgae. The glucose yields of the macroalgae *G. amansii*, *P. umbilicalis*, *M. pyrifera*, and *U. prolifera* were 75.24, 100.20, 67.80, and 142.87 mg/g DM, respectively. The cellulose conversion of macroalgae reached 84–98.59 %.

**Fig. 4:**
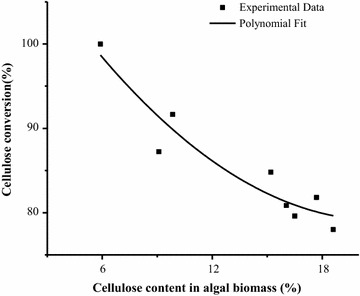
Correlation between cellulose content and cellulose conversion of algal biomass after thermal hydroxyl radical pretreatment

An exponential correlation was observed between the cellulose content of untreated algal biomass and treated algal biodegradability (Fig. 4). The algal species with high cellulose contents showed lower cellulose conversion than those with low cellulose contents. When the cellulose content in the algal biomass was lower than 20 %, the cellulose conversion rate can exceed 80 %. In general, algal biomass contains lower cellulose content (<20 %) than terrestrial biomass. Algal biomass with no lignin has relatively loose structure. The hydroxyl radical-aided thermal pretreatment uses hydroxyl radicals participating in the crystalline structural and hydrogen-bond disruptions. The milder reaction had no effect on high-value substances’ extraction and carbohydrate recovery. Therefore, the hydroxyl radical-aided thermal pretreatment is suitable for improving algal biomass digestibility. Terrestrial biomass usually has more than 30 % cellulose content and large amounts of hemicellulose and lignin, which are bound together in a complex structure. The ultrastructure of cellulose is compact due to the presence of covalent bonds, hydrogen bonding, and van der Waals forces. Due to the low concentration of hydroxyl radicals and short reaction time, the terrestrial biomass under this pretreatment condition does not have good saccharification results like algal biomass. For the terrestrial biomass with compact structure, this new pretreatment can be improved through extending the pretreatment time and increasing the hydroxyl radicals’ concentration.

Microalgal cells have abundant proteins, intracellular organic components, lipids, and other carbon-based compounds, which can easily leach during pretreatment. Therefore, microalgal biomass requires lower pretreatment conditions than macroalgal biomass [17]. However, different results were observed in the present study. The macroalgae under the same pretreatment conditions were more easily degraded than the microalgae. The microalgae had high contents of cellulose and hemicelluloses. These skeletal components are partially crystalline and are similar to the hemicelluloses of terrestrial plants [21, 22]. Macroalgae with lower cellulose content and looser structures need relatively milder pretreatment than microalgae. Therefore, the macroalgae had a higher cellulose digestibility than microalgae under similar conditions.

### Carbohydrate recovery of algal biomass after the novel pretreatment

Glucose and xylose were not detected in the liquid fraction, indicating that most sugars remained in the solid fraction after the pretreatment. Analysis of the eight types of algal biomasses revealed no changes in their chemical compositions compared to those before the pretreatment (Additional file 1: Table S1). This finding agrees with the earlier observation that no sugars were detected in the liquid fraction. The carbohydrate recovery of the eight types of algal biomasses reached 100 %, which was higher than those obtained under acid, hydrothermal, wet oxidation, and steam explosion pretreatments [7]. There has been a previous report that acid, hydrothermal, wet oxidation, and steam explosion pretreatments employed to algal biomass resulted in carbohydrate losses of about 24, 30, 50, and 31 g/100 g DM, respectively [7].

### Inhibitor analysis

Furfural and 5-HMF were not obtained from the eight types of algal biomass after the new pretreatment. This result suggests that the pretreatment in the present study is more suitable for algal biomass than conventional pretreatments. The lack of inhibitor formation coincides with the 100 % carbohydrate recovery, indicating that C6 and C5 sugars were not converted into inhibitors. The algal biomass after this pretreatment can be fermented to sugar or other high-value products.

Furfural and 5-HMF, which inhibit microbial metabolism, were observed after the conventional pretreatment. Furfural, 5-HMF, and acetic acid are formed from glucose and xylose under severe pretreatment conditions [18]. Furfural concentration increasess, while glucose and xylose concentrations decrease at pretreatment temperatures higher than 170 °C [18]. Hydrothermal and wet oxidation pretreatments of macroalgae yielded high concentrations of formic acid (0.7 and 1.8 g/100 g DM, respectively) and acetic acid (0.2 g/100 g and 1.0 g/100 g DM, respectively). A high amount of furfural (0.2 g/100 g DM) can be obtained after acid and hydrothermal pretreatments [11]. High amounts of furfural and formic acid can be formed through pretreatment with 7 % acid or at a high temperature (i.e., 180 °C) [6, 19].

### Structural changes in algal biomass using Fourier-transform infrared (FTIR) spectroscopy and X-ray diffraction (XRD) analysis

 FTIR and XRD analyses were conducted to determine the structural changes after the hydroxyl radical-aided thermal pretreatment (Table 3). The broad and strong absorbance within 4000–2995 cm^−1^ indicated the presence of hydrogen-bonded OH. The transmittance of the algal biomass after the pretreatment evidently increased, implying a decrease in the absorbance. The hydrogen-bond intensity (HBI) was calculated from the FTIR results. The hydroxyl radical-aided thermal pretreatment applied to the microalgae and macroalgae decreased the HBIs by 38.7–94.4 and 21.7–51.3 %, respectively. Simultaneously, the crystalline intensity (CrI) of the microalgae decreased by 30.8–81.0 %. However, the decrease in the crystalline intensity (CrI) of the macroalgae differed per species. The crystalline intensities of *U. prolifera* and *M. pyrifera* were both approximately 26 %. The crystalline intensity of *P. umbilicalis* slightly changed, whereas that of *G. amansii* decreased by 100 %. This result can be attributed to the variations in the composition of macroalgal biomass. However, the treated algal biomass had a higher digestibility than the untreated algal biomass. This finding coincides with the decreases in CrI and HBI in the algae.

**Table 3:** Glucose concentration released from *Macrocystis pyrifera* and *Ulva prolifera* under different conditions

Tri no.	Glucose released (mg/g DM)	Tri no.	Glucose released (mg/g DM)
J1	46.70 ± 0.044	H1	117.67 ± 0.079
J2	56.95 ± 0.042	H2	124.76 ± 0.082
J3	58.09 ± 0.051	H3	132.76 ± 0.067
J4	54.67 ± 0.032	H4	120.55 ± 0.043
J5	54.67 ± 0.031	H5	122.73 ± 0.045
J6	47.84 ± 0.027	H6	109.30 ± 0.035
J7	64.06 ± 0.061	H7	143.99 ± 0.047
J8	62.64 ± 0.064	H8	124.39 ± 0.068
J9	65.34 ± 0.057	H9	142.16 ± 0.064
J10	51.25 ± 0.044	H10	105.34 ± 0.057
J11	45.56 ± 0.024	H11	113.51 ± 0.078
J12	47.84 ± 0.027	H12	107.21 ± 0.057
J13	64.50 ± 0.052	H13	143.19 ± 0.045
J14	53.53 ± 0.042	H14	114.04 ± 0.041
J15	61.50 ± 0.046	H15	118.46 ± 0.049
Control	29.30 ± 0.038	Control	66.06 ± 0.027

### Mechanism of thermal hydroxyl radical-aided pretreatment

The highly effective action of the hydroxyl radicals in the newly developed process can be attributed to the abundant OH group in the polysaccharides of algal cell walls [20]. The hydrogen-bond network in algal cellulose is the bottleneck of algae enzymolysis. The improvement in algal biomass biodegradability after the hydroxyl radical-aided thermal pretreatment suggests that the pretreatment effectively deconstructed the algal cellulose hydrogen-bond network and crystalline structure. Therefore, this process can improve algal utilization.

**Fig. 5:**
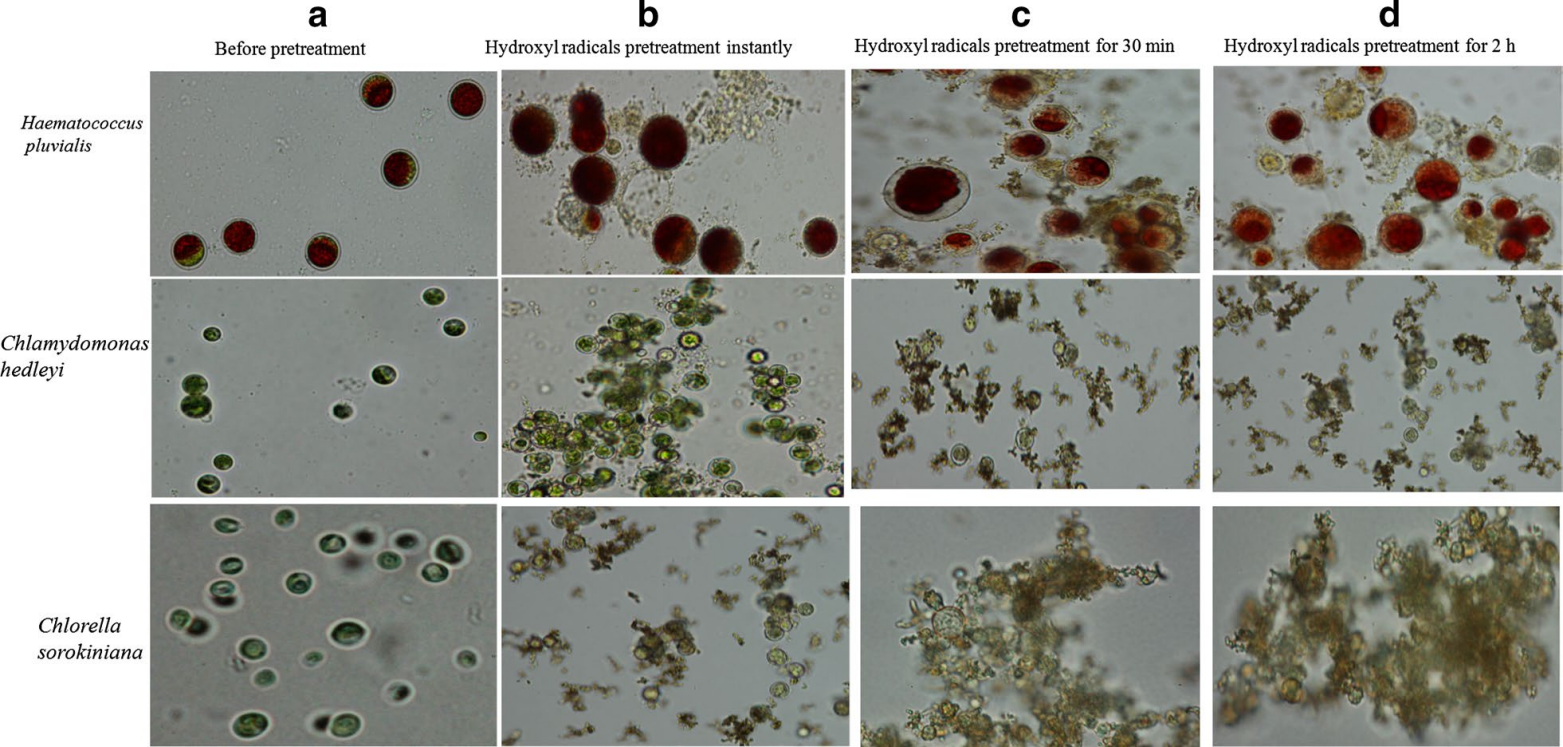
The microscopic images of **a** microalgae before pretreatment; **b** microalgae treated by hydroxyl radicals pretreatment instantly; **c** microalgae treated by hydroxyl radicals pretreatment for 30 min; **d** microalgae treated by hydroxyl radicals pretreatment for 2 h

Most carbohydrates in algae are incorporated within the cell wall [3]. Thus, cell wall disruption is required to release the available carbohydrates and to enable an effective saccharification. The hydroxyl radical-aided thermal pretreatment conducted in this study disrupted different algal cell walls under different concentrations of hydroxyl radicals. Microscopic pictures of the microalgae cells (*H. pluvialis; C. hedleyi; C. sorokiniana* as an example) before and after the hydroxyl radicals’ pretreatment process are shown in Fig. 5. The image before pretreatment shows the cells are clumped together, intact, and mostly spherical in shape. One the other hand, the microscopic image of the treated microalgae by hydroxyl radicals’ pretreatment instantly shows the edges of microalgae become blurred and the ruptured cell wall as evidenced by the broken cells. The microscopic image of the treated microalgae by hydroxyl radicals pretreatment for 2 h shows large cell debris mostly disappeared and a large number of small particles are present. The change from intact cells to broken cells after hydroxyl radicals’ pretreatment confirms the effect of the novel pretreatment in the disruption of microalgae cell wall.

The constituents of biopolymers in the algal biomass significantly differed from those of terrestrial biomass and also varied with algal species. The major polysaccharides in green algae are xyloarabinogalactans, such as xylans and mannans, which serve as partially crystalline skeletal components similar to the hemicelluloses of land plants and provide additional strength [21, 22]. These structural features possibly cause the difficult degradation of green algae. The cell walls of brown and red algae contain two layers, including an inner layer of cellulose and an outer layer of gelatinous substance. Brown algae contain alginates, laminarin, and mannitol as their principal polysaccharides in the outer layer, whereas red algae possess agar and carrageenan as their principal polysaccharides. The biopolymers from the different types of algal biomass after the hydroxyl radical-aided thermal pretreatment remained unchanged and undestroyed (data not shown). The new pretreatment had relatively milder reaction conditions than the conventional acid or alkali pretreatment. Basing on the characteristics of algal microstructure and the effect of hydroxyl radicals, we speculated the mechanism of thermal hydroxyl radical pretreatment. Hydroxyl radical-aided thermal pretreatment first removes alginate, mannitol, carrageenan, and agar in the outer layer of the algal biomass. Thus, the cellulose and hemicelluloses embedded inside the algal biomass become fully exposed. Adding FeSO_4_ and H_2_O_2_ creates a sufficient amount of ·OH. Moreover, the hydroxyl radical-aided thermal pretreatment requires a high temperature to accelerate the motion of hydroxyl radicals. Hydroxyl radicals rupture the algal cell walls by cleaving the intermolecular linkages between cellulose and cellulose, cellulose and hemicelluloses, and hemicelluloses and other components. Subsequently, the structural destruction of algal biomass facilitates cellulose degradation. The new pretreatment for algal biomass will be combined with algal biopolymer extraction to provide a novel method for the comprehensive utilization of algae.

This new pretreatment technology followed and simulated efficient conversion of natural biological way from nature. Simulate and reproduce the natural pretreatment process will help us profoundly understand the biomass biodegradation. The hydroxyl radical-aided thermal pretreatment selectively destruct algal cell walls and maximize the retention of cellulose and hemicellulose contents of algal biomass. The new pretreatment fundamentally solve the disadvantages of traditional pretreatment such as too strong reaction condition, low carbohydrate recovery, multi-inhibitors, and high-energy consumption. This new pretreatment may provide new insights into the low-cost and high-efficient transformation of algal biomass.

## Conclusion

Hydroxyl radical-aided thermal pretreatment was used as a novel method to disrupt the hydrogen bonds and cellulose crystallinity in algal biomass, which is beneficial to conversion of the carbohydrates in the algal cell wall into simple sugars by enzymolysis process. Overall, this method increased the amount of glucose released from the algal biomass. This method was proven suitable for the eight types of biomass. The new pretreatment requires low concentration of chemical solvents and milder temperature conditions, which can prevent the toxic and corrosive effects that typically result from conventional pretreatments. Moreover, the new method presents advantages over other pretreatment methods, including higher carbohydrate recovery, no inhibitor production, and lower-energy consumption. The new pretreatment development mimicking natural system could be useful for biochemical conversion of algal biomass to fuels and chemicals.

## Methods and materials

### Algal biomass

*Macrocystis pyrifera*, *U. prolifera*, *G. amansii*, and *P. umbilicalis* were purchased from QingDao MingYue Seaweed Group (Qingdao, China). *C. sorokiniana* (UTEX 1602) was obtained from the Culture Collection of Alga at the University of Texas (Austin, TX, USA). *H.* *pluvialis* was obtained from the Washington State University (Washington, USA)*. S.* *quadricanda* and *C. hedleyi* were collected from Bohai Bay, China [23]. All samples were dried at 65 °C for 24 h. The macroalgae were additionally milled to an average size of 80 mesh (0.22 mm) using a standard sieve. The screened samples were directly used in pretreatment studies.

### Hydroxyl radical-aided thermal pretreatment technologies

The pretreatment was conducted in 100 mL Erlenmeyer flasks with a total working volume of 50 mL. The reaction reactor was loaded with 2.5 g of dried algal biomass. The algal biomass was treated with optimal concentrations of H_2_O_2_ and FeSO_4_, and then stirred for 3–5 s. The algal biomass medium was thermally pretreated at different temperatures from 80 to 120 °C. The thermal pretreatment can enhance the functions of hydroxyl radicals. The reaction mixture was directly used for subsequent hydrolysis experiments.

### Experimental design

CCD was used to optimize the process parameters of the new algal pretreatment for glucose production. The parameters optimized in the hydroxyl radical-aided thermal pretreatment include reaction time, temperature, and H_2_O_2_ concentration (Table 4). The process variables in preliminary experiments were estimated through single factor experiments. Triplicate runs were conducted for each combination: H_2_O_2_ concentration (3.5–7.1 mM), temperatures (80–120 °C), and reaction times (15–45 min). The results from the experimental work were statistically analyzed using “Statistica 6.0” (StatSoft Inc., 2001) and “Minitab” (Minitab Inc.). The effect of each parameter was evaluated to determine the optimal parameters at which the highest glucose yield can be obtained.

**Table 4:** Comparison of the algal biomass digestibility, crystalline intensity, and hydrogen-bond intensity of different types of algal biomass before and after thermal hydroxyl pretreatment

	Microalgae								Macroalgae							
	*Chlorella sorokiniana*		*Scenedesmus quadricanda*		*Haematococcus* *pluvialis*		*Chlamydomonas hedleyi*		*Ulva prolifera*		*Porphyra umbilicalis*		*Gelidium amansii*		*Macrocystis pyrifera*	
	Before	After	Before	After	Before	After	Before	After	Before	After	Before	After	Before	After	Before	After
Enzymatic digestibility	47.73	81.82	49.90	80.85	3.63	79.61	10.20	80.27	23.09	84.79	14.57	91.64	39.23	87.23	44.69	98.59
Crystalline intensity	0.313	0.212	0.327	0.056	0.112	0.000	0.333	0.200	0.441	0.327	67.296	52.677	40.976	19.940	0.358	0.264
Hydrogen-bond intensity	92.628	56.750	46.482	2.600	37.149	31.948	164.188	61.342	220.970	81.526	0.388	0.378	0.260	0.000	102.065	58.407

### Analysis methods

#### Analysis of algal biomass composition

The carbohydrate in algal biomass was quantitatively analyzed according to the NREL Laboratory Analytical Procedures [28] for biomass using a two-step acid method [24]. Approximately 1 g (dry basis) of the samples was dispensed into 200 mL Erlenmeyer flasks. The samples were treated with 5 mL of 72 % (w/w) H_2_SO_4_ at 30 °C for 2.5 h and then stirred every 15 min with a glass stirring rod. The solutions were diluted with 181.7 mL of water and then autoclaved at 121 °C for 1 h. After autoclaving, the serum vials were cooled, and the samples were immediately centrifuged at 10,000×*g* for 20 min to remove solid particles. The supernatant was passed through a 0.22-µm filter. Glucose and xylose concentrations were determined using high-performance liquid chromatography (HPLC, Shimadzu, Kyoto, Japan) with a refractive index detector (Shimadzu) on an Aminex HPX-87H column (Bio-Rad, Hercules, CA, USA). The flow rate was 0.6 mL/min at 60 °C, and the mobile phase was 5 mM H_2_SO_4_. Glucan and xylan concentrations were calculated according to Eqs. (1) and (2), where factors of 0.9 and 0.88 reflect the weight losses in converting glucose to glucan and xylose to xylan, respectively [25].

1 $${\text{Glucan content }}\left( \% \right) = \frac{{{\text{Glucose released from acid hydrolysis (mg)}} \times 0. 9}}{\text{Samples weight (mg)}} \times 100\,\%$$

2 $${\text{Xylan content }}\left( \% \right) = \frac{{{\text{Xylose released from acid hydrolysis (mg)}} \times 0. 8 8}}{\text{Samples weight (mg)}} \times 100\,\%$$

Moisture content was measured by drying the samples at 105 °C in an oven until constant weight [26]. The content of intracellular proteins was determined using the Bradford method [29, 27]. The cells were disrupted through sonication in phosphate-buffered saline, and the supernatant was colored by mixing with the Bradford reagent (Sigma Chemical Co., St. Louis, MO, USA). Optical density was determined at 595 nm. Ash content was determined by heating the samples at 550 °C for 1 h. Crude lipid was extracted using the Soxhlet method with petroleum ether as the solvent [14].

#### Analysis of inhibitors

Furfural and 5-HMF were analyzed using HPLC (Shimadzu LC-20A, Kyoto, Japan) with a UV detector at a wavelength of 215 nm. The samples were diluted and passed through a 0.22 µm filter prior to analysis. 5-HMF and furfural were separated on an Aminex HPX-87H column (Bio-rad) at 35 °C using 5 mM H_2_SO_4_ as the eluent at 35 °C with a flow rate of 0.5 mL/min [28].

#### Analysis of hydrolyzates

The concentration of sugars in the liquid fraction of the enzymatic hydrolyzate was quantified using HPLC (Shimadzu, Kyoto, Japan) with a refractive index detector (Shimadzu). The samples were separated on an Aminex HPX-87H column (Bio-Rad, Hercules, CA, USA) using 5 mM H_2_SO_4_ as the mobile phase at 60 °C with a flow rate of 0.6 mL/min.

3 $${\text{Conversion of cellulose }}\left( \% \right) = \frac{{{\text{Glucose released from enzyme hydrolysis (mg)}} \times 0. 9}}{{{\text{Samples weight (mg)}} \times {\text{Glucan content (\% )}}}} \times 100\,\%$$

### FTIR spectroscopy and XRD analysis

The samples were dried at −20 °C for 24 h using a vacuum dryer (FD-IC-50, Beijing). IR spectra were determined using an FTIR 710 IR spectrophotometer (Nicolet, Madison, WI). A specific amount of the powder was dispersed in spectroscopic-grade KBr and then pressed into disks using ten tons of pressure for 1 min. A total of 100 scans with a 2 cm^−1^ resolution were signal averaged and then stored. The wave number was scanned within the range of 4000–400 cm^−1^. The ratio of absorbance at 4000–2995 cm^−1^ to those at 1337 cm^−1^ of C–OH in-plane stretching was introduced as an empirical criterion of HBI [29].

4 $${\text{HBI}} = \frac{{{\text{Absorbance}}\;(4000 - {\text{2995 {cm}}}^{ - 1}})}{{{\text{Absorbance\;(1337 cm}}^{ - 1} )}}$$

The overall crystallinity of the macroalgae subjected to the new pretreatment was examined through XRD on a Bruker D8 Advance Diffractometer with Cu Kα radiation (*λ* = 0.1541 nm) at 30 kV and 30 mA [24]. The samples were scanned, and the intensity was recorded in the 2*θ* range of 10°–80°.

To compare the intensity differences and determine the pretreatment effects, we calculated the CrI of the cotton fiber by referring to the diffraction intensities of the crystalline area and amorphous region using the following equation:

5 $${\text{CrI}} = \frac{{I_{002} - I_{\text{am}} }}{{I_{002} }}$$

where* I*_002_ is the intensity of the crystalline area of the plane at 2*θ* = 22.40, and *I*_am_ is the intensity of the amorphous region at 2*θ* = 18.70. *I*_002_ refers to both crystalline and amorphous intensities, whereas *I*_am_ only represents the background intensity.

### Enzymatic hydrolysis of pretreated algal biomass

The pretreated algal biomass and the control (untreated biomass) were subjected to enzymatic hydrolysis with cellulase at 50 °C for 72 h in triplicate. Cellulase from *Trichoder mareesei* ATCC 26921 was purchased from Sigma–Aldrich Co. Hydrolysis experiments were conducted in 50-mL Erlenmeyer flasks with a total working volume of 20 mL and a substrate concentration of 5 % (w/v). Enzyme loading was 15 FPU/g substrate. The reaction mixtures were supplemented with 0.5 % NaN_3_ to prevent microbial contamination. The samples were removed at regular intervals, and the supernatant was boiled to denature the enzyme and terminate its activity. The supernatant was passed through a 0.22-µm filter for glucose content analysis. After hydrolysis, the residues were separated from the liquid by centrifugation, decantation, and filtration. The sugar in the liquid was then analyzed.

## Additional file

10.1186/s13068-015-0372-2 Proximate analysis of dried algal biomass before and after hydroxyl radical-aided thermal pretreatment.

## Abbreviations

HPLC: high-performance liquid chromatography
DM: dry matter
HMF: hydroxymethylfurfural
FTIR: fourier transform infrared spectroscopy
XRD: X-ray diffraction
HBI: hydrogen-bond intensity
CCD: central composite design
CrI: crystallinity intensity
·OH: hydroxyl radicals
