# Supplementary material for: Hydroxyl radical-aided thermal pretreatment of algal biomass for enhanced biodegradability
Source: Biotechnol Biofuels. 2015 Nov 26;8:194. doi: 10.1186/s13068-015-0372-2 (PMC4681426; doi:10.1186/s13068-015-0372-2)
Supplement: Supplementary file 1 — 10.1186/s13068-015-0372-2 Proximate analysis of dried algal biomass before and after hydroxyl radical-aided thermal pretreatment. [file 13068_2015_372_MOESM1_ESM.docx]

|  | **Microalgae** | | |  | **Macroalgae** | | | | |
| --- | --- | --- | --- | --- | --- | --- | --- | --- | --- |
|  | *Chlorella sorokiniana* | *Scenedesmus quadricanda* | *Haematococcus pluvialis* | *Chlamydomonas*  *hedleyi* | *Ulva prolifera* | *Porphyra umbilicalis* | | *Gelidium amansii* | *Macrocystis pyrifera* |
| Cellulose (%)  Before pretreatment | 17.69±0.078 | 16.04±0.032 | 16.55±0.048 | 18.67±0.041 | 15.20±0.024 | | 9.84±0.011 | 9.09±0.013 | 5.90±0.010 |
| Cellulose (%)  After pretreatment | 17.58±0.071 | 16.24±0.062 | 16.59±0.041 | 18.77±0.049 | 15.10±0.014 | | 9.80±0.011 | 9.06±0.016 | 5.92±0.011 |
| Hemicellulose (%)  Before pretreatment | 4.99±0.014 | 0.82±0.049 | 14.42±0.034 | 1.45±0.012 | 3.61±0.014 | | 47.16±0.057 | 24.37±0.044 | 0.91±0.010 |
| Hemicellulose (%)  After pretreatment | 4.91±0.018 | 0.79±0.022 | 14.48±0.051 | 1.46±0.015 | 3.67±0.022 | | 47.22±0.078 | 24.44±0.059 | 0.93±0.014 |

Table S1
